# Alterations in the methylome of the stromal tumour microenvironment signal the presence and severity of prostate cancer

**DOI:** 10.1186/s13148-020-00836-2

**Published:** 2020-03-18

**Authors:** Mitchell G. Lawrence, Ruth Pidsley, Birunthi Niranjan, Melissa Papargiris, Brooke A. Pereira, Michelle Richards, Linda Teng, Sam Norden, Andrew Ryan, Mark Frydenberg, Clare Stirzaker, Renea A. Taylor, Gail P. Risbridger, Susan J. Clark

**Affiliations:** 1 Monash Partners Comprehensive Cancer Consortium, Monash Biomedicine Discovery Institute Cancer Program, Prostate Cancer Research Group, Department of Anatomy and Developmental Biology, Monash University, Clayton, VIC 3800 Australia; 2 Cancer Research Division, Peter MacCallum Cancer Centre, Melbourne, VIC 3000 Australia; 3 Sir Peter MacCallum Department of Oncology, The University of Melbourne, Parkville, VIC 3010 Australia; 4 Epigenetics Research Laboratory, Genomics and Epigenetics Theme, Garvan Institute of Medical Research, 384 Victoria St, Darlinghurst, Sydney, NSW 2010 Australia; 5 St. Vincent’s Clinical School, UNSW, Sydney, NSW 2052 Australia; 6 Invasion and Metastasis Laboratory, Cancer Division, The Kinghorn Cancer Centre, Garvan Institute of Medical Research, Darlinghurst, Sydney, NSW 2010 Australia; 7 TissuPath, Mount Waverley, VIC 3149 Australia; 8 Australian Urology Associates, Melbourne, VIC 3000 Australia; 9 Department of Urology, Cabrini Health, Malvern, VIC 3144 Australia; 10 Monash Partners Comprehensive Cancer Consortium, Monash Biomedicine Discovery Institute Cancer Program, Prostate Cancer Research Group, Department of Physiology, Monash University, Clayton, VIC 3800 Australia

**Keywords:** Prostate cancer, Tumour microenvironment, Cancer-associated fibroblast, Stroma, Methylation, Field effect, EPIC microarray

## Abstract

**Background:**

Prostate cancer changes the phenotype of cells within the stromal microenvironment, including fibroblasts, which in turn promote tumour progression. Functional changes in prostate cancer-associated fibroblasts (CAFs) coincide with alterations in DNA methylation levels at loci-specific regulatory regions. Yet, it is not clear how these methylation changes compare across CAFs from different patients. Therefore, we examined the consistency and prognostic significance of genome-wide DNA methylation profiles between CAFs from patients with different grades of primary prostate cancer.

**Results:**

We used Infinium MethylationEPIC BeadChips to evaluate genome-wide DNA methylation profiles from 18 matched CAFs and non-malignant prostate tissue fibroblasts (NPFs) from men with moderate to high grade prostate cancer, as well as five unmatched benign prostate tissue fibroblasts (BPFs) from men with benign prostatic hyperplasia. We identified two sets of differentially methylated regions (DMRs) in patient CAFs. One set of DMRs reproducibly differed between CAFs and fibroblasts from non-malignant tissue (NPFs and BPFs). Indeed, more than 1200 DMRs consistently changed in CAFs from every patient, regardless of tumour grade. The second set of DMRs varied between CAFs according to the severity of the tumour. Notably, hypomethylation of the *EDARADD* promoter occurred specifically in CAFs from high-grade tumours and correlated with increased transcript abundance and increased EDARADD staining in patient tissue. Across multiple cohorts, tumours with low *EDARADD* DNA methylation and high *EDARADD* mRNA expression were consistently associated with adverse clinical features and shorter recurrence free survival.

**Conclusions:**

We identified a large set of DMRs that are commonly shared across CAFs regardless of tumour grade and outcome, demonstrating highly consistent epigenome changes in the prostate tumour microenvironment. Additionally, we found that CAFs from aggressive prostate cancers have discrete methylation differences compared to CAFs from moderate risk prostate cancer. Together, our data demonstrates that the methylome of the tumour microenvironment reflects both the presence and the severity of the prostate cancer and, therefore, may provide diagnostic and prognostic potential.

## Background

In solid cancers, tumour formation changes the composition and phenotype of surrounding tissue. This creates the complex tumour microenvironment where different cell types, including cancer-associated fibroblasts (CAFs), interact with cancer epithelial cells [1]. CAFs are a heterogeneous population of cells that regulate the phenotype of prostate epithelial cells, including their tumourigenicity, proliferation, migration, invasion, differentiation and responsiveness to therapeutics [2–10]. CAFs also shape the tumour microenvironment by depositing extracellular matrix, promoting the infiltration of immune cells and stimulating angiogenesis [9, 11–13].

The functions of CAFs also evolve with cancer progression [14–17]. Indeed, changes in the histopathological features, gene expression profile and length of telomeres in the stroma have all been associated with poorer relapse-free or overall survival of men with prostate cancer [14, 15, 17–21]. The phenotype of CAFs is also enduring and does not rely on continuous interactions with epithelial cancer cells. This is demonstrated by primary cultures of patient-derived CAFs, which retain distinctive transcriptomic and proteomic profiles and ability to promote tumour progression, even when cultured without tumour epithelium [8, 10, 22, 23]. This stable phenotype is not due to genomic aberrations [24, 25]. Rather, we recently showed that CAFs harbour DNA methylation alterations compared to non-malignant prostate tissue fibroblasts (NPFs), particularly enriched at regulatory regions of the genome [24]. Other studies have also identified differential methylation of candidate genes and an altered repertoire of transcription factor binding sites in CAFs [6, 26, 27].

Although many epigenetic changes in CAFs have now been identified, their conservation between patients and association with prostate cancer aggressiveness is not clear. Therefore, in this study we compared the genome-wide methylation profiles of CAFs and NPFs from a larger cohort of men with primary prostate cancer, some of whom later developed advanced disease. Our results reveal two main sets of differentially methylated regions (DMRs) in CAFs. One set is CAF-specific, with methylation alterations that are remarkably consistent between CAFs and NPFs across all prostate cancers irrespective of grade. The second group of discrete methylation alterations was associated with tumour grade and patient outcome and may provide a potential source of prognostic biomarkers for prostate cancer.

## Results

### Profiling DNA methylation of prostate fibroblasts using EPIC arrays

**Fig. 1:**
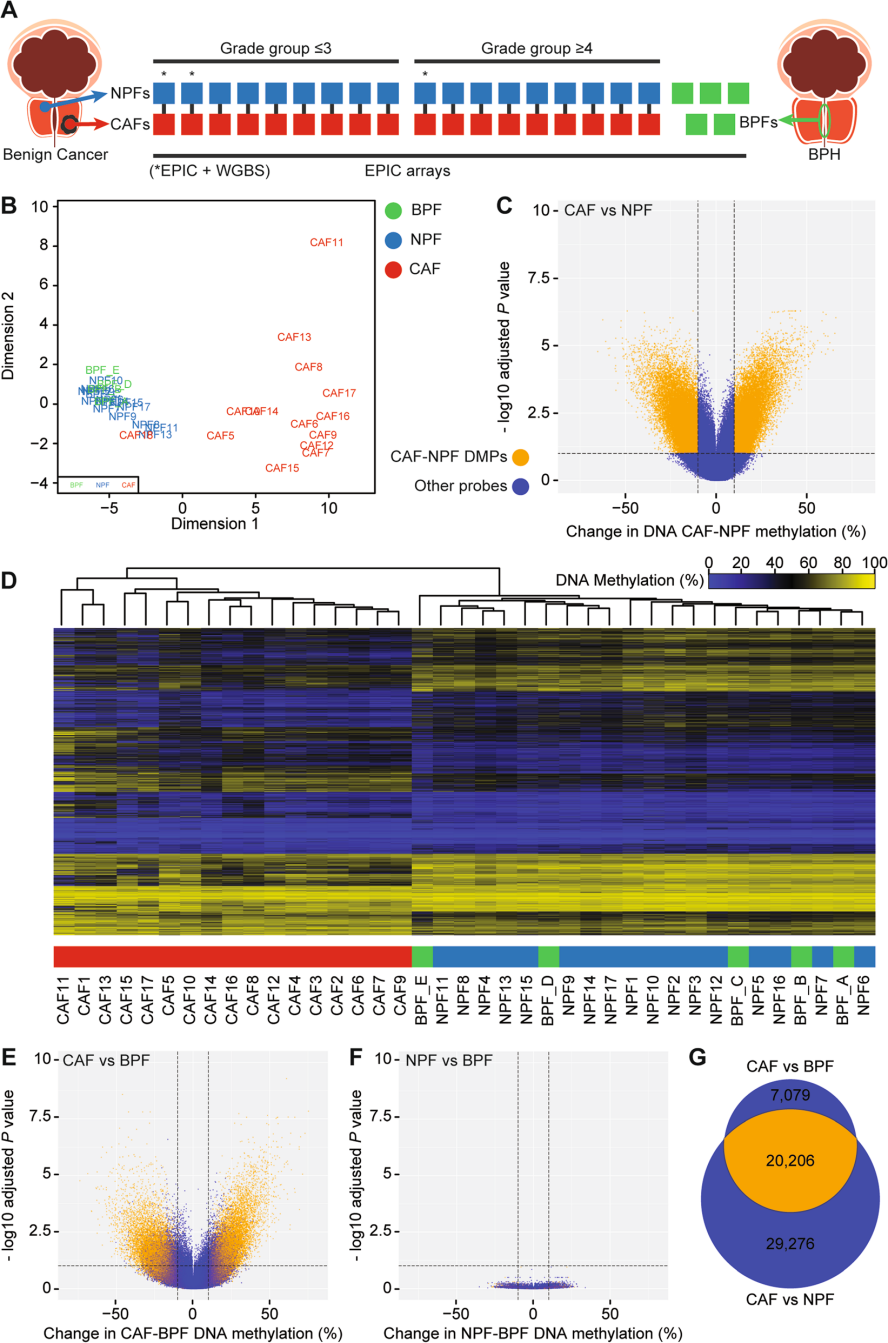
Prostate cancer-associated fibroblasts have distinctive changes in DNA methylation. **a** Schematic of the cohort of patient-derived fibroblasts analysed with EPIC arrays. Asterisks denote that WGBS data was available for three pairs of CAFs and NPFs. **b** MDS plot of the 1000 most variably methylated CpGs in EPIC array data showing clear separation of CAFs from NPFs and BPFs in patients 4–17; however, CAF18 clustered with NPFs and BPFs. **c** Volcano plot of differentially methylated positions (DMPs) in CAFs versus NPFs (patients 4–17). DMPs are shown in orange, while other probes are in blue. For all volcano plots, dotted lines indicate > 10% change in methylation and −log_10_ adjusted *P* value > 1 (adjusted *p* value > 0.1). **d** Dendrogram and heat map from unsupervised hierarchical clustering of the EPIC CAF-DMRs showing clear separation of CAFs from NPFs and BPFs. **e** and **f** Volcano plots of DMPs in CAFs versus BPFs and NPFs versus BPFs. DMPs from CAFs versus NPFs (panel **c**) are shown in orange. **g** Venn diagram showing the overlap between DMPs in CAFs versus NPFs compared to CAFs versus BPFs

We used Infinium MethylationEPIC BeadChips (EPIC arrays) to examine the genome-wide DNA methylation profile of early passage CAFs and NPFs from eighteen men (*n* = 36 samples) with either moderate or high-grade prostate cancer (Fig. 1a). The fibroblasts were patient-matched, with CAFs from tumour tissue and NPFs from distant benign tissue from a contralateral region and/or different anatomical zone of the same prostate, most often the transition zone (Additional File 1: Table S1). A pathologist verified the histology of all patient tissues. Nine patients had moderate-grade prostate cancer, defined as grade group ≤ 3 (GG ≤ 3; Gleason score 6–7), and nine patients had high-grade disease, defined as grade group ≥ 4 (GG ≥ 4; Gleason 8–10). The GG ≥ 4 patients had significantly higher primary tumour volume, shorter relapse-free survival and greater incidence of distant metastases (Table 1 and Additional File 1: Table S1). For cross-platform validation, we included three cases with published whole genome bisulfite sequencing (WGBS) data (Additional File 1: Table S1) [24]. To enable more thorough comparison of the methylation profiles of fibroblasts from benign and malignant tissue, we also included unmatched benign prostate tissue fibroblasts (BPFs) from patients undergoing transurethral resection of the prostate for benign prostatic hyperplasia (*n* = 5 men). These patients had no evidence of prostate cancer after at least 5 years follow-up.

**Table 1:** Clinical features and follow-up of patients with ≤ GG3 and ≥ GG4 prostate cancer

	**≤ GG3**	**≥ GG4**	***P*value**
Patients, no.	9	9	
Age, median (range)	68 (53–72.4)	65 (60–74)	0.8249^a^
Gleason Grade Group, no. (%)			
GG2	5 (56%)	0	
GG3	4 (44%)	0	
GG4	0	1 (11%)	
GG5	0	8 (89%)	
Clinical features, median (range)			
PSA ng/mL	6.7 (4-11)	7 (4.3–22.6)	0.3072^a^
Tumour volume	4 (0.7-7.1)	19.7 (0.7–30.2)	**0.0103**^a^
Clinical features, no. (%)			
Pathologic T stage 2	2 (22%)	2 (22%)	1.0^b^
Pathologic T stage 3	7 (78%)	7 (78%)	
Positive margins	6 (67%)	3 (33%)	0.6372^c^
Extra-prostatic extension	7 (78%)	7 (78%)	1.0^c^
Seminal vesicle invasion	3 (33%)	7 (78%)	0.1534^c^
Lymph node metastases at diagnosis	0	4 (44%)	0.0824^c^
Patient follow-up^d^, no. (%)			
Disease relapse^e^	2 (25%)	8 (89%)	**0.0030**^e^
Metastasis	1 (13%)	7 (78%)	**0.0152**^c^
Castration-resistant prostate cancer	0 (0%)	3 (33%)	0.2059^c^
Death from prostate cancer	0 (0%)	1 (11%)	1.0^c^

^a^Unpaired *T* test with Welch’s correction

^b^The Fisher exact test comparing the proportion of patients with T2 versus T3 disease

^c^The Fisher exact test comparing the proportion of patients with or without each clinical feature

^d^Follow-up information was unavailable for one GG ≤ 3 patient, so *n* = 8

^e^Disease relapse includes biochemical or clinical recurrence, HR = 6.937 (1.738–27.68), log rank test

The EPIC array methylation data showed technical reproducibility with WGBS. DNA methylation levels were highly correlated across the 796,222 CpG sites common to both platforms for three patient-matched pairs of CAFs and NPFs (patients 1–3, Pearson correlation 0.84–0.87) (Additional File 2: Figure S1a) [24]. As we previously reported, there was no evidence of global hypomethylation in CAFs versus NPFs (Additional File 2: Figure S1a) [24]. Nevertheless, CAFs clearly separated from NPFs in the first dimension of multidimensional scaling (MDS) plots of both datasets (Additional File 2: Figure S1b-c). Furthermore, there was excellent concordance in previously identified DMRs in CAFs versus NPFs (CAF-DMRs) between the two platforms (Additional File 2: Figure S1d), based on 3384 regions with probes on the EPIC array. For example, hypermethylated DMRs in the *TBX3* gene were consistently detected in each patient by both EPIC arrays and WGBS (Additional File 2: Figure S1e). Altogether, this demonstrates the accuracy of the EPIC platform for measuring DNA methylation values and differential methylation in this study.

### CAFs have distinct methylation profiles from NPFs and BPFs

To examine the methylation profile of BPFs, NPFs and CAFs, we generated an MDS plot of the EPIC methylation data, excluding the three patients used for technical validation (i.e., patients 4–18 only) (Fig. 1b). CAFs formed a separate group from NPFs and BPFs in the first dimension of the analysis, confirming their distinct DNA methylation profiles across patients. CAFs were also more dispersed than NPFs and BPFs in the second dimension of the plot. The plot suggests some patient-to-patient epigenetic variation in CAFs, but minimal differences in methylation among NPFs and BPFs.

Unexpectedly, CAF18 clustered with NPFs and BPFs rather than CAFs (Fig. 1b). To further analyse CAF18, we used an in vitro co-culture assay that measures the ability of fibroblasts to induce morphological changes in prostate epithelial cells [8, 28]. Unlike other CAFs, CAF18 did not induce significant phenotypic changes in prostate epithelial cells compared to its patient-matched NPF (Additional File 1: Table S1 and Additional File 2: Figure S2a & b). Given that CAF18 was atypical in both DNA methylation and the functional assay, we excluded this patient from further analyses. We speculate that CAF18 was originally misclassified as a CAF possibly due to poor sampling of the patient’s tumour tissue.

### Identifying novel differentially methylated regions

Since the remaining CAFs formed a separate cluster in the MDS plot (Fig. 1b), we performed a new genome-wide analysis to identify specific regions of differential methylation. We excluded the three patients (patients 1–3) previously analysed with WGBS [21]. This revealed ~ 50,000 significantly differentially methylated positions (DMPs) between CAFs and NPFs (Fig. 1c, adjusted *P* value < 0.1 and absolute methylation difference > 10%). These DMPs could be further grouped into DMRs: 2369 hypermethylated and 3038 hypomethylated with more than 10% difference in methylation in CAFs versus NPFs (Additional File 1: Table S2 & S3). These regions are herein referred to as EPIC CAF-DMRs. Unsupervised clustering of samples using the methylation of probes within the EPIC CAF-DMRs separated all CAFs from both NPFs and BPFs (Fig. 1d). Notably, 2059 of the hypermethylated regions (87%) and 2501 of the hypomethylated regions (82%) were not previously reported with WGBS.

### NPFs and BPFs have negligible differences in DNA methylation

Based on the MDS plot and EPIC CAF-DMRs (Fig. 1b, d), we found that NPFs are more similar to unmatched BPFs than they are to their patient-matched CAFs. To further compare each set of prostatic fibroblasts, we performed genome-wide analysis of differential methylation using limma. Strikingly, we identified 27,285 DMPs in CAFs versus BPFs (Fig. 1e), but no significant DMPs in NPFs versus BPFs (Fig. 1f; adjusted *P* value < 0.1 and absolute methylation difference > 10%). Therefore, we conclude that NPFs and BPFs share very similar methylomes, despite being from different patients with different prostatic diseases. Furthermore, the DMPs in CAFs versus BPFs were largely the same as those between CAFs versus NPFs (Fig. 1e, g).

### CAF-DMRs are consistent across patients

**Fig. 2:**
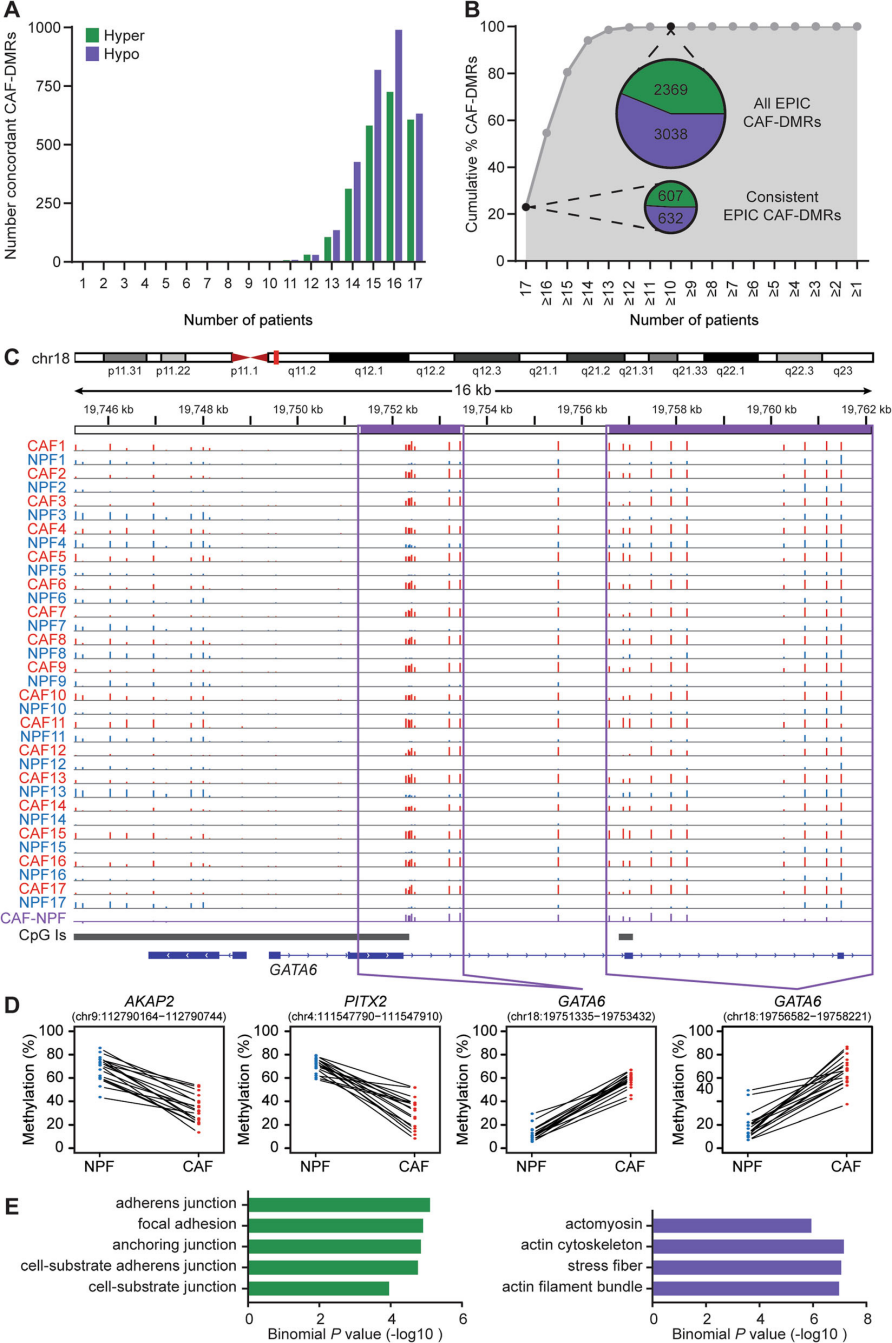
Consistently differentially methylated regions across patients in CAFs versus NPFs. **a** Graph showing the number of EPIC CAF-DMRs that are concordantly differentially methylated in the expected direction in each number of patients. **b** Graph showing the cumulative percentage of EPIC CAF-DMRs that are concordantly differentially methylated in the expected direction in each number of patients. Inset pie charts show the number of concordant EPIC CAF-DMRs in 17/17 patients (22.0% of DMRs) and 10/17 patients (100% of DMRs). **c** EPIC data for the *GATA6* gene for each NPF (blue) and CAF (red). The average difference in DNA methylation in CAFs compared to NPFs is shown in purple. The height of each vertical line represents the percentage of DNA methylation at each CpG site. Purple boxes show the site of two EPIC CAF-DMRs. **d** Graphs showing DNA methylation levels in each NPF and CAF for representative hypomethylated (*AKAP2* and *PITX2*) and hypermethylated (*GATA6*) consistent EPIC CAF-DMRs. Lines connect each patient-matched pair of fibroblasts. For each sample, the percentage of DNA methylation is averaged across CpG sites within each DMR. **e** Plots showing −log_10_ binomial *P* values of pathways within the cellular content category that were enriched in GREAT analysis of hypermethylated (green) and hypomethylated (purple) consistent EPIC CAF-DMRs

To further examine the EPIC CAF-DMRs, we determined how consistent they were across patients. The majority of hypermethylated and hypomethylated EPIC CAF-DMRs were present in most patients (Fig. 2a). Indeed, 80% of EPIC CAF-DMRs showed concordant methylation differences in at least 15 of the 17 patients, and all were shared by at least 10 of the patients (Fig. 2b). Furthermore, 1239 ‘consistent EPIC CAF-DMRs’ had concordant methylation differences in all 17 patients (607/2369 (26%) of hypermethylated and 632/3038 (21%) of hypomethylated EPIC CAF-DMRs; Fig. 2a, b and Additional File 1: Tables S2 and S3). These consistent EPIC CAF-DMRs encompassed 1.6% of the CpG sites assayed by the EPIC array (hypermethylated DMRs span 2161 probes, hypomethylated DMRs span 10,744 probes). The differentially methylated genes included *GATA6*, with two hypermethylated DMRs in all 17 CAFs compared to their matched NPFs (Fig. 2c, d). Conversely, *PITX2* and *AKAP2* had hypomethylated DMRs in all patient’s CAFs (Fig. 2d). We also confirmed that there were significant correlations between DNA methylation and mRNA abundance for candidate EPIC CAF-DMRs (Additional File 2: Figure S3A-B).

To investigate the possible functional importance of the 1239 consistent EPIC CAF-DMRs, we used the Genomic Regions Enrichment of Annotation Tool (GREAT) [29]. We observed enrichment of terms related to cell adhesion (focal adhesion, cell-substrate adherens junction, stress fibre, actin filament bundle), as well as ligand activated cell signalling, including TGFβ, insulin and PDGF signalling pathways (Fig. 2e and Additional File 1: Table S4). The remarkable concordance of the methylation changes at this large set of consistent EPIC CAF-DMRs across patients, coupled with their association with genes in biologically relevant pathways, suggests their likely importance in defining the identity and functions of CAFs in prostate cancer.

### *EDARADD* is hypomethylated in grade group ≥ 4 CAFs

**Fig. 3:**
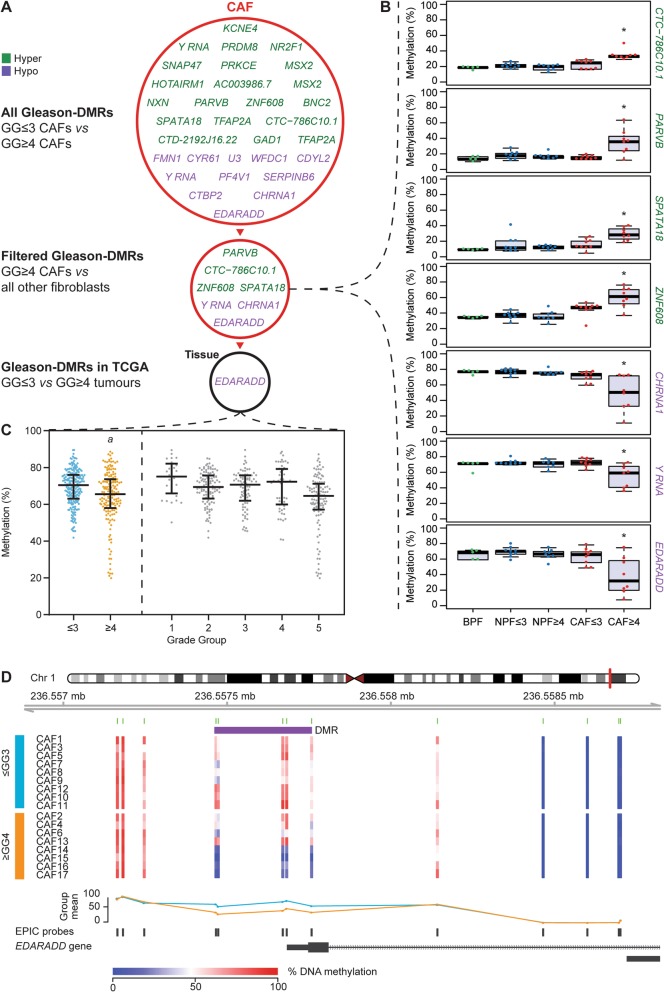
*EDARADD* is hypomethylated in CAFs from high-grade group prostate cancer. **a** Schematic of genes proximal to Gleason-DMRs in CAFs from GG ≤ 3 versus GG ≥ 4 prostate cancer. Gleason-DMRs that are hypermethylated in GG ≥ 4 CAFs are shown in green, while Gleason-DMRs that are hypomethylated in GG ≥ 4 CAFs are shown in purple. Seven of these Gleason-DMRs were also differentially methylated in GG ≥ 4 CAFs versus all other groups of fibroblasts (see panel **b**). Of these Gleason-DMRs, *EDARADD* was also significantly differentially methylated in GG ≥ 4 versus GG ≤ 3 tissues from TCGA (see panel **c**). **b** Boxplots showing DNA methylation of Gleason-DMRs in different groups of fibroblasts. Each dot represents a different fibroblast sample (*P < 0.05 One-way ANOVA GG ≥ 4 CAF vs all other groups). **c** Plot of *EDARADD* DNA methylation levels in patient tissue samples from TCGA. Samples are arranged as GG ≤ 3 versus GG ≥ 4 prostate cancer (^*a*^*P* = 8.3 × 10^−5^, diff = − 5.2%, Mann-Whitney test) and as individual grade groups. Each dot represents a different patient, with lines indicating median and ± IQR. **d** Schematic of the *EDARADD* Gleason-DMR showing the levels of DNA methylation at each CpG site in each CAF (blue = low methylation; red = high methylation). The trend lines show the average methylation status of GG ≤ 3 CAFs (light blue) versus GG ≥ 4 CAFs (orange). The location of the Gleason-DMR is shown in purple

In addition to the set of DMRs that distinguish CAFs from NPFs, we next examined whether there is a set of DMRs that identify patients with more aggressive tumours. We compared the DNA methylation profiles of CAFs from GG ≤ 3 (*n* = 9) versus GG ≥ 4 tumours (*n* = 8) and used DMRcate to identify regions with absolute methylation differences greater than 10%, which we termed Gleason-DMRs. We found 31 Gleason-DMRs; only four of which were previously identified as CAF-DMRs [24] (Fig. 3a and Additional File 1: Table S5). To verify the cell-type specificity of the Gleason-DMRs, we used ANOVA models to compare methylation levels at Gleason-DMRs between the five sets of fibroblasts (BPFs, GG ≤ 3 NPFs, GG ≥ 4 NPFs, GG ≤ 3 CAFs and GG ≥ 4 CAFs). Seven of the Gleason-DMRs were significantly different between GG ≥ 4 CAFs and all other fibroblast groups (*P* < 0.05, Fig. 3a, b). Of these, the *EDARADD* Gleason-DMR had the greatest methylation difference in GG ≥ 4 CAFs versus other fibroblasts (mean methylation difference of 26%; Additional File 1: Table S5).

Next, to assess their potential application as prognostic biomarkers we examined whether the seven Gleason-DMRs in CAFs could also be detected in whole patient tumour tissue, which contains heterogeneous cell types. We analysed the Infinium Methylation450 BeadChip (450 K array) data from 392 prostate cancer samples in TCGA. We found that only the *EDARADD* Gleason-DMR was significantly differentially methylated between GG ≤ 3 (*n* = 226) versus GG ≥ 4 tumours (*n* = 166) (Fig. 3a, c). A sequential decrease in DNA methylation of the *EDARADD* Gleason-DMR was also apparent from low to high-grade group samples (Fig. 3c). EDARADD is an adaptor in the EDAR pathway, which regulates the development of ectodermal tissues [30]. *EDARADD* is also differentially expressed in prostate and lung cancer [31, 32], which is notable since the Gleason-DMR lies in the potential gene promoter (Fig. 3d). Based on these observations, we examined the association between *EDARADD* methylation and gene expression levels and high risk prostate cancer in more detail.

### *EDARADD* is differentially expressed in high-grade prostate cancer

**Fig. 4:**
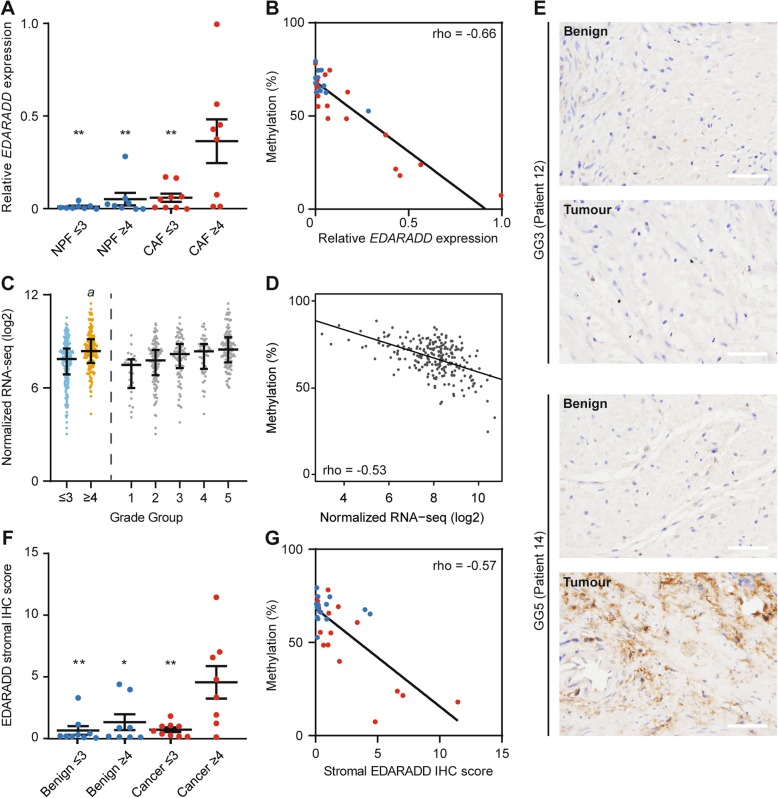
*EDARADD* expression is increased in high-grade prostate cancer and correlated with DNA methylation. **a** Plot showing the average expression of *EDARADD* (± SEM) in each group of NPFs (blue) and CAFs (red). There was significantly higher mRNA abundance in ≥ GG4 CAFs versus each other fibroblast group (**P < 0.01 One-way ANOVA with Tukey post hoc analysis). **b** Scatter plot showing the significant negative correlation between EPIC data for *EDARADD* DNA methylation and qRT-PCR data for *EDARADD* mRNA abundance (Spearman correlation, *P* < 0.0001). Each dot represents a different fibroblast sample. **c** Plot of RNA-seq data showing higher *EDARADD* expression in ≥ GG4 versus ≤ GG3 prostate cancer specimens from TCGA (*b* logFC between GG1-3 vs GG4-5 = 1.57, genome-wide adjusted *P* = 6.9 × 10^−07^, generalized linear model using edgeR). **d** Scatter plot of matching *EDARADD* 450K DNA methylation data and RNA-seq data from TCGA showing a significant negative correlation (Spearman correlation, *P* = 3.2 × 10^17^). **e** Representative images of immunohistochemistry (IHC) for EDARADD in matched benign and tumour tissues. Scale bars equal 50 μm. **f** Plot of the average EDARADD stromal IHC score (± SEM) in each group of patient tissues. There was significantly higher EDARADD staining in ≥ GG4 tumours versus ≤ GG3 tumours and benign samples (*P < 0.05, **P < 0.01 One-way ANOVA with Tukey post hoc analysis). **g** Scatter plot showing the significant negative correlation between *EDARADD* DNA methylation in fibroblasts and the stromal EDARADD IHC score in matching patient tissues (Spearman correlation, *P* = 0.0006)

Since the *EDARADD* Gleason-DMR lies in a potential promoter for this gene, we assessed *EDARADD* mRNA and protein levels and their correlation with methylation. *EDARADD* mRNA levels were significantly higher in CAFs from grade group ≥ 4 tumours compared to all other groups of fibroblasts (BPFs, GG ≤ 3 NPFs, GG ≥ 4 NPFs and GG ≤ 3 CAFs), as measured using qPCR (Fig. 4a). Accordingly, there was a significant negative correlation between *EDARADD* mRNA abundance and DNA methylation at the Gleason-DMR across CAFs and NPFs (Fig. 4b). We observed the same pattern of *EDARADD* mRNA levels in patient tissues from TCGA, with significantly higher expression in high-grade group tumours (Fig. 4c) and a significant negative correlation with DNA methylation of probes in the Gleason-DMR (Fig. 4d).

We also used immunohistochemistry, with appropriate controls, to examine EDARADD protein levels in the original patient samples from which the CAFs and NPFs were established (Fig. 4e and Additional File 2: Figure S4a-c). Stromal EDARADD staining was significantly higher in grade group ≥ 4 tumours compared to other patient samples (Fig. 4f) and negatively correlated with *EDARADD* methylation in the matching fibroblasts (Fig. 4 g). EDARADD was also expressed in the epithelium, but with no significant difference in staining between grade groups (Additional File 2: Figure S4d). We noted that a subset of patients have particularly pronounced changes in *EDARADD* methylation, expression and stromal staining compared to other patients. This is evident in the frequency histograms showing wider ranges of values for CAFs compared to NPFs, and longer tails of values for tumour compared to benign tissue from the TCGA cohort (Additional File 2: Figure S5a-e).

### *EDARADD* methylation is associated with age in non-malignant prostate samples

Intriguingly, the precise region of the *EDARADD* Gleason-DMR, specifically EPIC probe cg09809672 (chr1:236,557,682, hg19), is known to be gradually hypomethylated with age in human blood and saliva samples [33, 34]. Indeed, we observed a significant negative correlation between cg09809672 methylation and patient age in NPFs and normal prostate tissues from TCGA (Additional File 2: Figure S6a-b), but this trend was much more subtle in CAFs and tumour tissues from TCGA (Additional File 2: Figure S6c-d). This concurs with a previous study showing a weaker association between DNA methylation and chronological age in cancer tissues compared with healthy tissues [35].

We also investigated whether the hypomethylation of *EDARADD* in the high-grade group samples might signify an accelerated aging phenotype, based on an established DNA methylation signature frequently observed in cancer [35]. However, we found no difference in the DNA methylation aging signature between NPFs and CAFs or grade group ≤ 3 CAFs and grade group ≥ 4 CAFs (Additional File 2: Figure S6e). Nor was there a relationship between the aging signature and *EDARADD* methylation across all fibroblasts (Additional File 2: Figure S6f). Thus, *EDARADD* is gradually hypomethylated with age in non-malignant prostate samples, but the significant decrease in *EDARADD* methylation in high-grade CAF samples is not linked to a more general aging phenotype in these cells. This is consistent with the known weakness of the relationship between an accelerated aging signature and tumour grade [35].

### *EDARADD* methylation and expression are associated with poor clinical features and patient outcomes

Since *EDARADD* methylation and expression are associated with grade group in CAFs and tumour tissue, we examined whether there was any association with other clinical features or patient outcomes in several published prostate cancer cohorts. In each cohort, to capture the subset of patients with epigenetic changes in *EDARADD*, we compared patients in the bottom quartile of *EDARADD* methylation or top quartile of *EDARADD* expression to the rest of the cohort. In TCGA, patients in the lowest quartile of *EDARADD* methylation or highest quartile of expression had significantly higher grade group (Table 2). They also had significantly higher pathologic tumour stage and incidence of positive lymph nodes but no difference in age at diagnosis (Table 2).

**Table 2:** Clinical features of TCGA patients based on *EDARADD* expression and methylation

**TCGA DNA methylation**	**Bottom 0.25**	**Top 0.75**	***P*value**
Patients, no.	97	290	
Age, median (range)	62 (47–75)	62 (44–78)	0.5339^a^
Gleason Grade Group, no. (%)			
GG1	1 (1%)	26 (9%)	**< 0.0001**^b^
GG2	18 (19%)	96 (33%)	
GG3	18 (19%)	63 (21%)	
GG4	16 (16%)	36 (12%)	
GG5	44 (45%)	69 (24%)	
Clinical features, no. (%)			
Pathologic T stage 2	24 *of 96* (25%)	115 *of 286* (40%)	**0.0072**^c^
Pathologic T stage 3+	72 (75%)	171 (60%)	
Lymph node involvement	33 *of 91* (36%)	36 *of 240* (13%)	**< 0.0001**^d^
Patient follow-up			
Relapse, no of events	44	26	
Log rank test^e^			**0.0095**
Cox model^e^			**0.0167**
**TCGA RNA levels**	**Top 0.25**	**Bottom 0.75**	***P*value**
Patients, no.	95	285	
Age, median (range)	62 (46–78)	62 (44–77)	0.7623^a^
Gleason Grade Group, no. (%)			
GG1	1 (1%)	26 (9%)	**< 0.0001**^**b**^
GG2	20 (24%)	94 (33%)	
GG3	20 (24%)	61 (21%)	
GG4	13 (15%)	36 (13%)	
GG5	41 (48%)	68 (24%)	
Clinical features, no. (%)			
Pathologic T stage 2	16 *of 94* (17%)	120 *of 281* (43%)	**< 0.0001**^**c**^
Pathologic T stage 3+	78 (83%)	161 (57%)	
Lymph node involvement	28 *of 90* (31%)	34 *of 223* (15%)	**0.0026**^**d**^
Patient follow up			
Relapse, no of events	42	27	
Log rank test^f^			**0.0054**
Cox model^f^			**0.0005**

Sample numbers are based on the availability of clinical, methylation (387 samples) and expression (380 samples) data. Numbers in italics denote sample numbers where data was not available for some cases

^a^Unpaired *T* test with Welch’s correction

^b^Chi-squared test for trend

^c^The Fisher exact test comparing the proportion of patients with T2 versus T3 disease

^d^Chi-squared test

^e^Log rank HR = 0.48 (0.28–0.84), Cox model HR = 0.10 (0.012–0.66)

^f^Log rank HR 1.96 (1.13–3.39), Cox model HR = 1.41 (1.16–1.72)

**Fig. 5:**
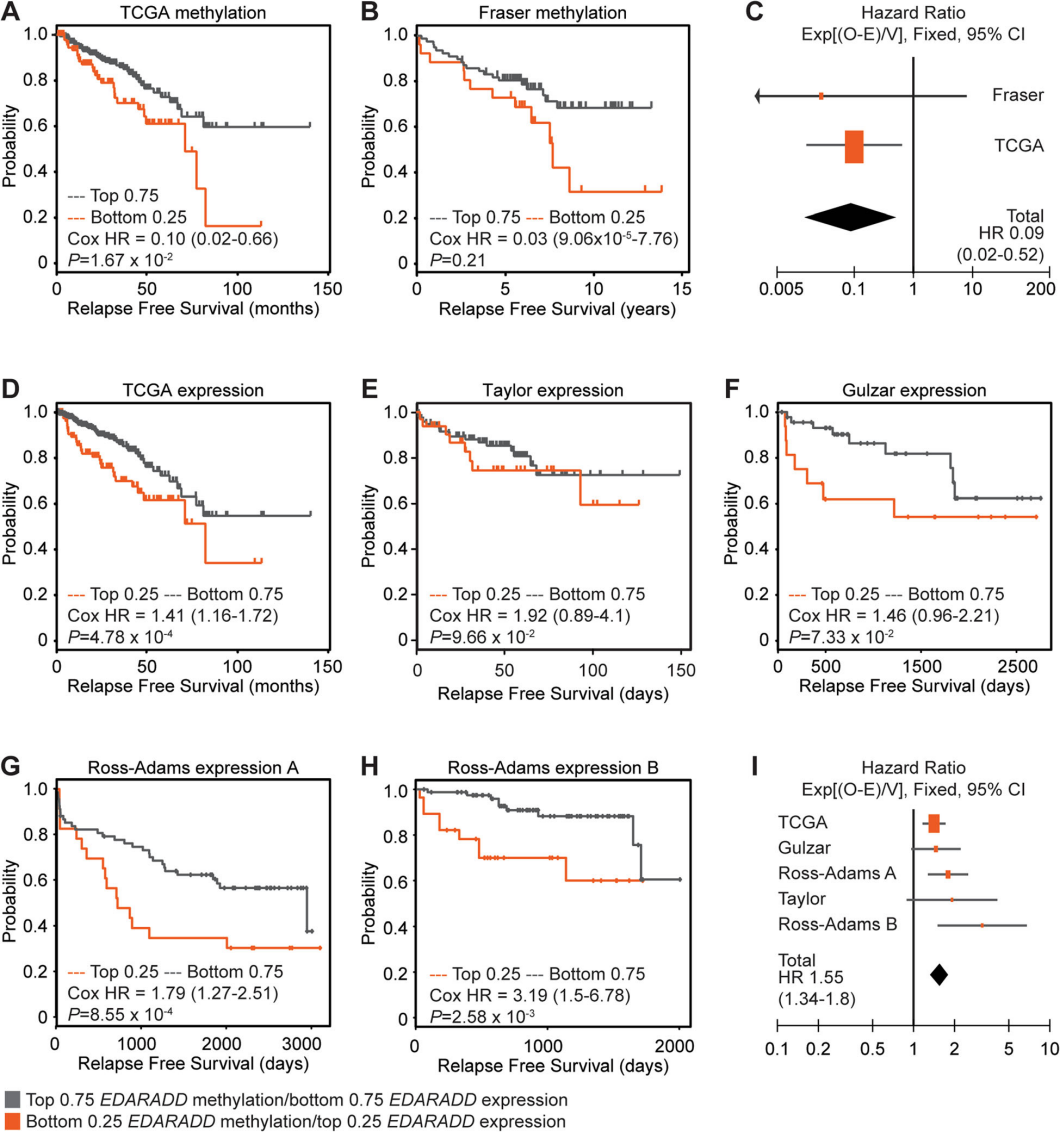
*EDARADD* methylation and expression are associated with poor relapse-free survival in prostate cancer cohorts. **a**, **b** Kaplan Meier plots of relapse free survival for patients in the lowest quartile of *EDARADD* methylation (bottom 0.25, orange) versus the rest of each cohort (top 0.75, grey). **c** Forest plot showing the Cox hazard ratios (± 95% CI) for relapse free survival based on *EDARADD* methylation and a meta-analysis of both methylation datasets (Heterogeneity: Chi^2^ = 0.09, df = 1 (*P* = 0.76); *I*^2^ = 0%; Test for overall effect: *Z* = 3.14 (*P* = 0.002)). **d**–**h** Kaplan Meier plots of relapse free survival for patients in the highest quartile of *EDARADD* expression (top 0.25, orange) versus the rest of each cohort (bottom 0.75, grey) for the TCGA and Fraser datasets. **i** Forest plot showing the Cox hazard ratios (± 95% CI) for relapse-free survival based on *EDARADD* expression and a meta-analysis of all methylation datasets (Heterogeneity: Chi^2^ = 5.45, df = 4 (*P* = 0.24); I^2^ = 27%; Test for overall effect: *Z* = 5.74 (*P* < 0.00001))

We also examined differences in relapse-free survival using Kaplan Meier curves and Cox models of *EDARADD* methylation or expression as continuous variables. Low *EDARADD* methylation was significantly associated with shorter relapse-free survival in TCGA patients (Fig. 5a and Table 2). We observed the same trend in methylation in the Fraser cohort, even though it is restricted to low-moderate risk prostate cancer (Fig. 5b) [36]. A meta-analysis confirmed that the association between *EDARADD* hypomethylation and poor relapse-free survival was significant across both datasets (Fixed effect model, *Z* = 3.14, *P* = 0.002, Fig. 5c).

Consistent with the inverse correlation between *EDARADD* methylation and expression, high *EDARADD* expression was significantly associated with poor relapse-free survival among patients in TCGA (Fig. 5d). There was the same trend in four other patient cohorts (Fig. 5e–h), reaching significance in both datasets from Ross-Adams and colleagues [37–39]. A meta-analysis of all five cohorts confirmed that higher *EDARADD* expression is significantly associated with shorter relapse-free survival (Fixed effect model, *Z* = 5.74, *P* < 0.00001, Fig. 5i). Finally, the levels of *EDARADD* methylation and expression in CAFs, and stromal staining in matched tumour tissues, were all significantly associated with poor relapse-free survival in our cohort (Additional File 2: Figure S7), consistent with the overall difference in survival between the grade group ≤ 3 and grade group ≥ 4 patients (Table 1). Therefore, epigenetic changes in *EDARADD* are consistently associated with adverse clinical features and poor relapse-free survival in men with localised prostate cancer.

## Discussion

Tumourigenesis is associated with genome-wide DNA methylation alterations of cells within the tumour microenvironment, including CAFs [24]. Yet, how consistent these changes are across individual patients and whether they vary based on clinical features is unknown. Therefore, we assessed the DNA methylation profiles of prostatic fibroblasts from carefully validated patient samples spanning moderate- to high-grade prostate cancer. We found that a set of differently methylated regions accurately distinguished CAFs from patient-matched NPFs and non-matched BPFs, including a newly identified set of CAF-NPF DMRs with remarkable consistency across patients. We also identified DMRs associated with higher grade group disease, including at the promoter of *EDARADD*, which was associated with shorter relapse-free survival of patients.

Differences in the methylomes of CAFs or tumour stroma have been observed in primary cell cultures, patient tissue and mouse models of prostate cancer, although often in small numbers of samples [6, 24, 26, 40, 41]. To further examine the technical and biological reproducibility of DMRs, we used EPIC arrays to analyse genome-wide DNA methylation in a larger cohort of patients. There was strong cross-platform validation between EPIC arrays and WGBS, which both separated CAFs from NPFs. Furthermore, we identified numerous DMRs in CAFs versus NPFs, including 1239 CAF-DMRs that were detected in CAFs from every patient. This is remarkable given the heterogeneity of CAFs and the diversity of our cohort, with CAFs isolated from primary cancers with different grade groups, tumour stages, treatment outcomes and presumably genomic aberrations. These DMRs may be useful to validate primary cultures of CAFs and NPFs in future studies. Pathway analyses with the DMRs highlighted the importance of cell adhesion, cell morphology and the PDGF and TGFβ pathways, in concordance with previous studies [2, 42]. Thus, the common functional features of CAFs may include the mechanisms for attaching to the surrounding microenvironment and signalling to other cell types. Overall, the consistent CAF-DMRs imply that key molecular aspects of the identity of CAFs are preserved across prostate cancer samples.

In addition to comparing matched CAFs and NPFs, we examined unmatched BPFs to investigate whether NPFs bear traces of a cancer field effect [43]. The concept of field cancerisation is of particular interest in prostate cancer, because many patients have multifocal disease. Histologically normal tissues adjacent to tumour foci can also exhibit cytomorphological, transcriptional, genomic and epigenomic alterations close to tumour foci [16, 17, 44, 45]. Although some DNA methylation alterations have been reported in more distant regions of non-malignant prostate tissue [41, 46–50], we found negligible differences in our study between the DNA methylation profiles of NPFs and BPFs. Further analysis of CAF-DMRs in samples at varying distances from the tumour may define how far the cancer field effect extends and whether it is variable with tumour grade.

Previous studies have reported progressive changes in the histopathology and transcriptome of tumour stroma from low- to high-grade prostate cancer, so we hypothesised that there may also be changes in DNA methylation [17, 19]. Indeed, we identified a limited number of DNA methylation alterations associated with tumour grade. Notably *EDARADD* showed the greatest difference in methylation in grade group ≥ 4 CAFs. Loss of methylation at the *EDARADD* promoter is particularly interesting from a clinical perspective, since it is correlated with increased mRNA expression and stromal staining. EDARADD is an intracellular adaptor protein in the ectodysplasin pathway, activating downstream NFκB signalling when the EDA ligand binds to the EDAR receptor [30]. The ectodysplasin pathway fine-tunes the development of ectodermal tissues such as hair, teeth, sweat glands and mammary glands [51]. Patients with mutations in this pathway, including of *EDARADD*, have ectodermal dysplasias with malformations of ectodermal tissues. *EDARADD* is also associated with aging, through hypomethylation of cg09809672 [33, 34]. We observed that this CpG site is indeed hypomethylated with aging in non-malignant prostate tissue; however, the pronounced hypomethylation of *EDARADD* in high-grade tumour samples was not associated with a more widespread aging signature in this tissue. Although the function of EDARADD and the ectodysplasin pathway in the prostate is unknown, it is possible that it influences the paracrine interactions between stroma and epithelium, since it is expressed in both cell types and regulates the expression of Wnt, FGF and chemokines in other tissues [30, 51].

Further investigation of the role of EDARADD in tumour progression is warranted, given its association with poor patient outcomes across multiple cohorts. In this study, we observed that DNA methylation and gene expression levels of *EDARADD* are associated with tumour grade, stage, lymph node metastasis and relapse-free survival. *EDARADD* has also been linked to tumour severity in other studies. Shahabi and colleagues showed that *EDARADD* expression is upregulated in prostate cancer tissue from patients who develop clinical recurrence [31]. In addition hypomethylation and increased expression of *EDARADD* in CAFs and patient tissue is associated with poor overall survival in non-small cell lung cancer [32].

## Conclusions

This study identified a set of DNA methylation alterations that are specific to CAFs and shared across patients regardless of tumour grade. These shared epigenetic changes potentially encode the phenotypic differences between CAFs and NPFs. We also demonstrated that *EDARADD* methylation and expression correlate with clinical features and patient outcomes, indicating that specific epigenome changes in CAFs reflect the features of the adjacent tumour. Importantly, *EDARADD* represents a potential prognostic biomarker to detect the severity of the tumour based on the molecular features of the surrounding microenvironment.

## Methods

### Patient tissue

Samples of fresh prostate tissue (*n* = 41, Additional File 1: Table S1) were collected from 23 patients undergoing radical prostatectomy (Table 1) or transurethral resection of the prostate (TURP) with written informed consent according to human ethics approval from Monash University (2004/145), Cabrini Hospital (03-14-04-08) and Epworth Hospital (53611). To isolate CAFs from radical prostatectomy specimens, the location of the tumour was determined using biopsy reports and palpation. The prostate was cut to expose the tumour, and frozen sections were examined by a board-certified pathologist to confirm that the area contained approximately 80% prostate cancer. Approximately, 200–1000 mg of tissue was then dissected from this site. To isolate patient-matched NPFs, benign tissue was obtained from a distant region of the same radical prostatectomy specimen, typically from a different side and zone of the prostate. Frozen sections were used to confirm the lack of tumour cells in the benign tissue. The prostate gland was then reassembled and processed for routine histopathology. BPFs were isolated from TURP chips from men with benign prostatic hyperplasia, but no diagnosis of prostate cancer for at least 5 years after the specimens were collected. Patient clinical features and follow-up were collected by the Melbourne Urological Research Alliance (MURAL). Gleason scores were converted to grade groups (GG) as described [52]. Relapse-free survival was calculated as the time from radical prostatectomy to biochemical relapse (two consecutive and rising serum PSA measurements > 0.2 ng/mL) [53] or clinical relapse [54], whichever occurred first.

### Cell culture

Primary cultures of prostatic fibroblasts were established and validated as previously described [8, 43]. Briefly, fresh patient tissue was chopped into small pieces, approximately 2 mm^3^, and two or three pieces were retained for histology. Fibroblasts were only included in this study if a pathologist confirmed that these pieces of fixed tissue had the correct pathology: benign for BPF and NPF tissues and tumour for CAF tissues. The rest of each tissue was digested overnight at 37 °C in RPMI containing 10% fetal calf serum (FCS), 25 mM HEPES, 100 U/mL penicillin, 100 mg/mL streptomycin, 0.5 mg/mL Amphotericin B, 100 mg/mL gentamicin, 225 U/mL Collagenase Type I and 125 U/mL Hyaluronidase Type II (Sigma-Aldrich) as previously described [43]. Cells were then seeded in RPMI containing 5% FCS, penicillin/streptomycin, 1 nM testosterone (Sigma-Aldrich) and 10 ng/mL bFGF (Millipore), which selects for the growth of fibroblasts versus other prostatic cell types. Cells were grown at 37 °C in a humidified incubator with 5% O_2_ and 5% CO_2_. Early passage cultures of fibroblasts (median P4, range P2–7) were used for subsequent experiments.

### Microarray genome-wide DNA methylation analysis

DNA was extracted from fibroblast samples with the DNeasy kit (Qiagen) with on-column RNase A digestion. DNA (500 ng) from 18 patient-matched CAF-NPF pairs and 5 BPF samples was treated with sodium bisulphite using the EZ-96 DNA methylation kit (Zymo Research, CA, USA). DNA methylation was quantified using the Illumina Infinium HumanMethylationEPIC (EPIC) BeadChip (Illumina, CA, USA) run on an Illumina HiScan System (Illumina, CA, USA) using the manufacturer’s standard protocol.

Raw intensity data (IDAT) files were imported into the R environment (version 3.1.1) [55] using the *minfi* package (version 1.20.2) [56]. Each sample passed all quality control steps. The data correctly predicted all patients to be male and control single nucleotide polymorphism (SNP) probes correctly paired the patient-matched CAFs and NPFs. Data was then normalised with background correction. Poor quality probes with a detection *P* value > 0.01 in at least 10% of the samples were removed. At least 99% of probes passed this step. Poor quality probes with a detection *P* value > 0.01 in less than 10% samples were considered undetected. Next, to reduce the risk of false discoveries, we removed probes that mapped to multiple locations or overlapped SNPs, as previously described [40]. The resulting dataset comprised 808,100 CpG sites. β values were calculated from unmethylated (U) and methylated (M) signal [M/(U + M + 100)] and ranged from 0 to 1 (0 to 100% methylation). The co-ordinates of all CpG sites were defined using the hg19 human genome assembly.

### WGBS data extraction

To compare EPIC and WGBS data, we used in-house CAF and NPF WGBS sequencing data that was generated and processed as previously described [24]. All raw and processed WGBS data is publically available at NCBI Gene Expression Omnibus (GEO) (www.ncbi.nlm.nih.gov/geo) under accession number GSE86833. We used the *getMeth* function in R package bsseq [57] to extract CAF-NPF WGBS data for patients 1, 2 and 4 at the 796,222/808,100 CpG sites overlapping the EPIC probes in our dataset. To compare EPIC and extracted WGBS methylation data, we used base package functions in R to produce scatterplots, perform Pearson correlation analysis, and output bedGraph files of the data for visualisation in the IGV genome browser [58].

### EPIC array statistical analysis

For initial visualisation of the EPIC data, multidimensional scaling plots were generated using the ‘mdsPlot’ function in the *minfi* Bioconductor package (version 1.20.2) [56]. We then performed differential methylation analysis between novel CAF versus NPF (*n* = 14 vs *n* = 14), CAF versus BPF (*n* = 17 vs *n* = 5), NPF versus BPF (*n* = 17 vs *n* = 5) and between Gleason grade groups (*n* = 8 GG ≤ 3 CAFs vs *n* = 7 GG ≥ 4 CAFs). In each case, β values were transformed using logit transformation: *M* = log2(β/(1−β)). We used the limma Bioconductor package [59] to identify DMPs between sample groups with adjusted *p* value cut-off of < 0.1. DMPs were visualised as volcano plots using the ggplot2 R package [60]. The R package DMRcate [61] was used to identify DMRs, with DMP *p* value cut-offs of FDR < 0.05 for CAF versus NPF and *p<* 0.0001 for GG ≤ 3 versus GG ≥ 4. DMRs were defined as regions with a maximum of 1000 nucleotides between consecutive probes and a minimum of 2 CpG sites, a methylation change > 10% and we applied Benjamini-Hochberg correction for multiple testing. DMRs were annotated for proximity with genetic features using the ‘annotateRegions’ function implemented in the R package aaRon (https://github.com/astatham/aaRon). DMRs were visualised as heat maps with dendrograms using the heatmap.2 function in the gplots R package [62], and bedGraph files of the data were generated for visualisation in the IGV genome browser [58]. ‘Consistent EPIC CAF-DMRs’ were identified by subtraction of methylation differences between each patient-matched CAF and NPF, and GREAT was used to analyse the functional significance of these DMRs [29].

To establish the cell-type specificity of the Gleason-DMRs, we used base package functions in R to perform one-way ANOVA and Tukey post hoc tests to compare Gleason-DMRs in GG ≥ 4 CAFs to all other fibroblast groups. This analysis was performed on a single methylation value for each Gleason-DMR per sample, obtained by calculating the mean methylation across all probes in the region and plotted using the beeswarm package in R [63].

To determine the DNA methylation age of each fibroblast sample, we uploaded β values from EPIC array data to the DNA Methylation Age Calculator (https://dnamage.genetics.ucla.edu/) [35].

### Cellularised matrix co-culture model

A cellularised matrix co-culture model was used as previously described with some modifications [8, 28]. Briefly, CAFs and NPFs were seeded in 24 well plates at 1.5 × 10^4^ cells/well and cultured for 5–8 days to yield a dense monolayer with extensive extracellular matrix deposition. RWPE-1 cells (American Type Culture Collection) [64] were maintained in keratinocyte serum-free medium supplemented with 5 ng/mL epidermal growth factor (Gibco), 50 μg/mL bovine pituitary extract (Gibco), 100 U/mL penicillin and 100 mg/mL streptomycin at 37 °C, 5% CO_2_. For co-cultures, RWPE-1 cells were pre-stained with CellTracker Green CMFDA (Invitrogen), seeded on top of the fibroblasts at 1.5 × 10^4^ cells per well, and cultured at 37 °C, 5% CO_2_, 5% O_2_. After 24 h, cellularised matrix co-cultures were fixed with 4% paraformaldehyde for 12 min and then washed with phosphate-buffered saline.

Cellularised matrix co-cultures were imaged at 488 nm and with brightfield microscopy using a Nikon C1 Inverted Eclipse 90i confocal microscope with a × 20 objective lens. 2D quantitative analysis of RWPE-1 cell morphology was performed using ImageJ (NIH) as previously described [8]. Briefly, a maximum intensity projection was obtained of the green-labelled RWPE-1 cells, then a Gaussian Blur filter (σ: 2) was applied, followed by thresholding, and the watershed step to obtain the outlines of the cells. The shape factor, cell area, cell length and standard deviation of orientation of these outlines were then calculated in 8 random fields per co-culture with an average of 58 cells/field (range 20–140).

### Quantitative RT-PCR

Total RNA was isolated from prostatic fibroblasts using the RNeasy Kit (QIAGEN) with an on-column DNaseI treatment. A mixture of RNA from human prostatic fibroblasts, epithelial cells and immune cells was pooled and used as a universal prostate control. Each sample (500 ng) was reverse transcribed into cDNA using the Superscript III First Strand Synthesis System (Invitrogen) according to the manufacturer’s instructions. Primer sequences are listed in Additional File 1: Table S6. Gene expression was quantified using Power SYBRTM Green Master Mix (ThermoFisher Scientific) and a Mx3000P qPCR System with MxPro Software (Stratagene). The relative mRNA abundance of the candidate genes compared to the universal prostate cancer control was calculated using the ΔΔCt method and the geometric mean of the three reference genes (*GAPDH*, *HPRT1*, *RPLPO*).

### Immunohistochemistry

Small pieces of tissue were retained from the specimens used to establish fibroblast cultures. These samples were formalin-fixed and paraffin embedded. Sections were stained with a rabbit anti-EDARADD antibody (1 μg/mL, HPA018836, Sigma) or rabbit IgG control (1 μg/mL, Dako) using a Leica BOND-MAX-TM autostainer with BondTM epitope retrieval 1 and the Bond Refine Detection Kit (Leica). Slides were imaged using a ScanScope AT Turbo slide scanner (Aperio). Regions of stroma and epithelium in each tissue were circled separately using the ImageScope analysis software (Aperio), and staining was quantified with the positive pixel count v9 algorithm. Positive staining was defined as the percentage of strong positive pixels (intensity limit 0–100) versus the total number of pixels analysed per sample.

### Analysis of 450K methylation datasets

Prostate adenocarcinoma (PRAD) 450K methylation data was downloaded from The Cancer Genome Atlas (TCGA) Data Portal website (http://tcga-data.nci.nih.gov/tcgafiles) and processed as described in [24], giving 414,133 CpG sites from 437 samples (of which 392 were tumour tissue). Gleason scores were converted to grade groups as described [52]. We identified probes that overlapped the seven CAF GG ≥ 4 Gleason-DMRs and calculated the mean methylation of probes within each region for each sample. The difference in methylation β values between tissues from *n* = 226 GG ≤ 3 versus *n* = 166 GG ≥ 4 prostate cancers was determined with a *t* test.

The 450K methylation data from the Fraser cohort was downloaded from the NCBI GEO database with accession GSE84043 [36, 65]. IDAT files were imported into the R environment (version 3.1.1) [55] using the *minfi* package (version 1.20.2) [56]. Data quality was checked with plots derived from control probes on the array. Data was then normalised with background correction. Poor quality probes with a detection *P* value > 0.01 in at least 10% samples were removed. Next, to reduce the risk of false discoveries we removed probes that mapped to multiple locations or overlapped SNPs [66]. The resulting dataset comprised 444,775 CpG sites. β values for the 160 tumour samples were calculated from unmethylated (U) and methylated (M) signal [M/(U + M + 100)] and ranged from 0 to 1 (0 to 100% methylation). β values were averaged across technical replicates, leaving 104 unique patient samples for analysis. Corresponding patient clinical data was obtained from Supplementary Table 1 in [36].

### Analysis of TCGA RNAseq data

TCGA PRAD processed RNA-seq V2 data (level 3) was downloaded from the TCGA Data Portal website (http://tcga-data.nci.nih.gov/tcgafiles) on 19th April 2016. We extracted the samples matching the earlier TCGA PRAD 450K methylation data (described above—tumour *n* = 385/392). Spearman correlation analysis was used to assess the relationship between DNA methylation at probes within the *EDARADD* Gleason-DMR and *EDARADD* gene expression. Additionally, differential gene expression between Gleason grade groups (*n* = 226 GG ≤ 3 versus *n* = 159 GG ≥ 4) was calculated genome-wide using edgeR [67] and log fold change and Bonferroni adjusted *P* value extracted for *EDARADD*.

### Analysis of patient clinical features in public datasets

Clinical data for the TCGA PRAD samples (corresponding to the methylation and expression data above) were downloaded from cBioPortal [68, 69] (TCGA—Provisional) on 13th August 2018. Samples with known disease-free status were included for further analyses (*n* = 387 for 450K methylation, *n* = 380 for RNAseq). Clinical 450K methylation data for the Fraser cohort [36] (GSE84043) and Affymetrix array expression data from the Taylor cohort [39] (GSE21032) were downloaded from the NCBI GEO database [65]. The Taylor dataset was processed as previously described [24]. Samples in the bottom quartile of *EDARADD* DNA methylation or top quartile of *EDARADD* expression were compared to the rest of the samples in each dataset. Relapse-free survival was visualised using Kaplan-Meier plots and was defined as biochemical relapse or disease-free survival as reported for each dataset. Cox proportional hazards models were used to calculate hazard ratios and *P* values using the R survival 2.39 package [70]. Additional survival analyses were performed with PROGgene2 [71] using data from the Gulzar cohort [37] (GSE40272) and two cohorts from Ross-Adams and colleagues (GSE70768 and GSE70769) [38].

### Meta-analyses

Data from Cox proportional hazard models were used for meta-analyses of the association between relapse-free survival and *EDARADD* methylation and expression. The *O-E* and *V* values for each dataset were calculated as previously described [72]. The Review Manager version 5.3 software was used for meta-analyses [73]. The statistical model was Exp[(O-E)/Var], the statistical method was fixed effect and the effect measure was hazard ratios. The resulting Forest plots were ordered by the effect size of each dataset.

## Supplementary information

**Additional File 1.** Supplementary Tables S1, S2, S3, S4, S5, S6.

**Additional File 2.** Figures S1, S2, S3, S4, S5, S6, S7.

## Data Availability

The data generated as part of this study are available from NCBI Gene Expression Omnibus (GEO) (www.ncbi.nlm.nih.gov/geo) under accession number GSE115413. The datasets analysed during the current study are available in the following open access repositories: GEO, https://www.ncbi.nlm.nih.gov/geo/ (GEO accession number: GSE21032, GSE84043, GSE86833) TCGA, https://cancergenome.nih.gov/ PROGgene, http://watson.compbio.iupui.edu/chirayu/proggene/database/ (Datasets GSE40272, GSE70768, GSE70769).

## Abbreviations

450K array: Infinium Methylation450 BeadChip
BPF: Benign prostate tissue fibroblast
CAF: Cancer-associated fibroblast
DMR: Differentially methylated region
*EDARADD*: EDAR associated death domain
EPIC array: Illumina Infinium HumanMethylationEPIC BeadChip
FCS: Fetal calf serum
GEO: Gene Expression Omnibus
GG: Grade group
MDS: Multidimensional scaling
MURAL: Melbourne Urological Research Alliance
NPF: Non-malignant prostate tissue fibroblast
RP: Radical prostatectomy
RT-PCR: Reverse transcription polymerase chain reaction
TCGA: The Cancer Genome Atlas
TURP: Transurethral resection of the prostate
WGBS: Whole genome bisulfite sequencing
