# Supplementary material for: Alterations in the methylome of the stromal tumour microenvironment signal the presence and severity of prostate cancer
Source: Clin Epigenetics. 2020 Mar 18;12:48. doi: 10.1186/s13148-020-00836-2 (PMC7081708; doi:10.1186/s13148-020-00836-2)
Supplement: Supplementary file 2 — Additional File 2. Figures S1, S2, S3, S4, S5, S6, S7. [file 13148_2020_836_MOESM2_ESM.pdf]

Additional File 2

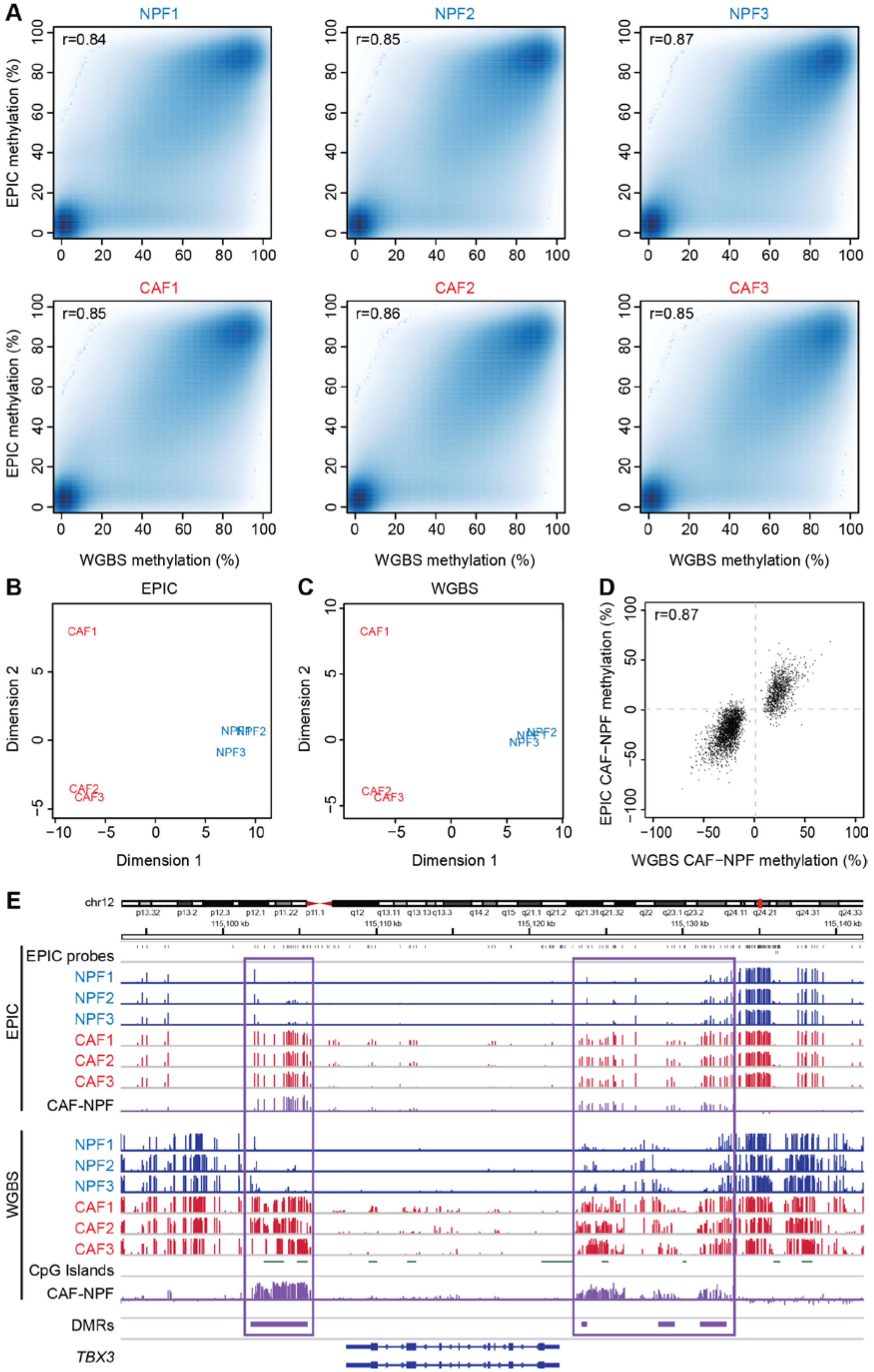

Figure S1

Comparison of methylation values on the EPIC platform with WGBS. **a** Scatter plots of DNA methylation between cross-platform replicates of WGBS and EPIC arrays at CpG sites interrogated by both platforms. r = Pearson correlation coefficient. All samples had a correlation *P* value $<2.2\text{x}10^{-16}$. **b-c** MDS plots showing clear separation of CAFs and NPFs based on the 1000 most variably methylated CpGs in (**b**) EPIC array and (**c**) WGBS data. **d** Cross-platform validation of EPIC and WGBS methylation data for the 45% of WGBS CAF-DMRs overlapped by probes on the EPIC array. Each dot represents the mean difference in DNA methylation (n=3 pairs) averaged across each DMR. Pearson's r =0.87, *P* $<2.\ 2\text{x}10^{-16}$. **e** EPIC and WGBS data for the *TBX3* gene for each NPF (blue) and CAF (red). The average difference in DNA methylation in CAFs compared to NPFs is shown in purple. The height of each vertical line represents the percentage of DNA methylation at each CpG site. The location of EPIC probes is shown in grey. The purple boxes highlight large regions of CAF hypermethylation measured on both platforms.

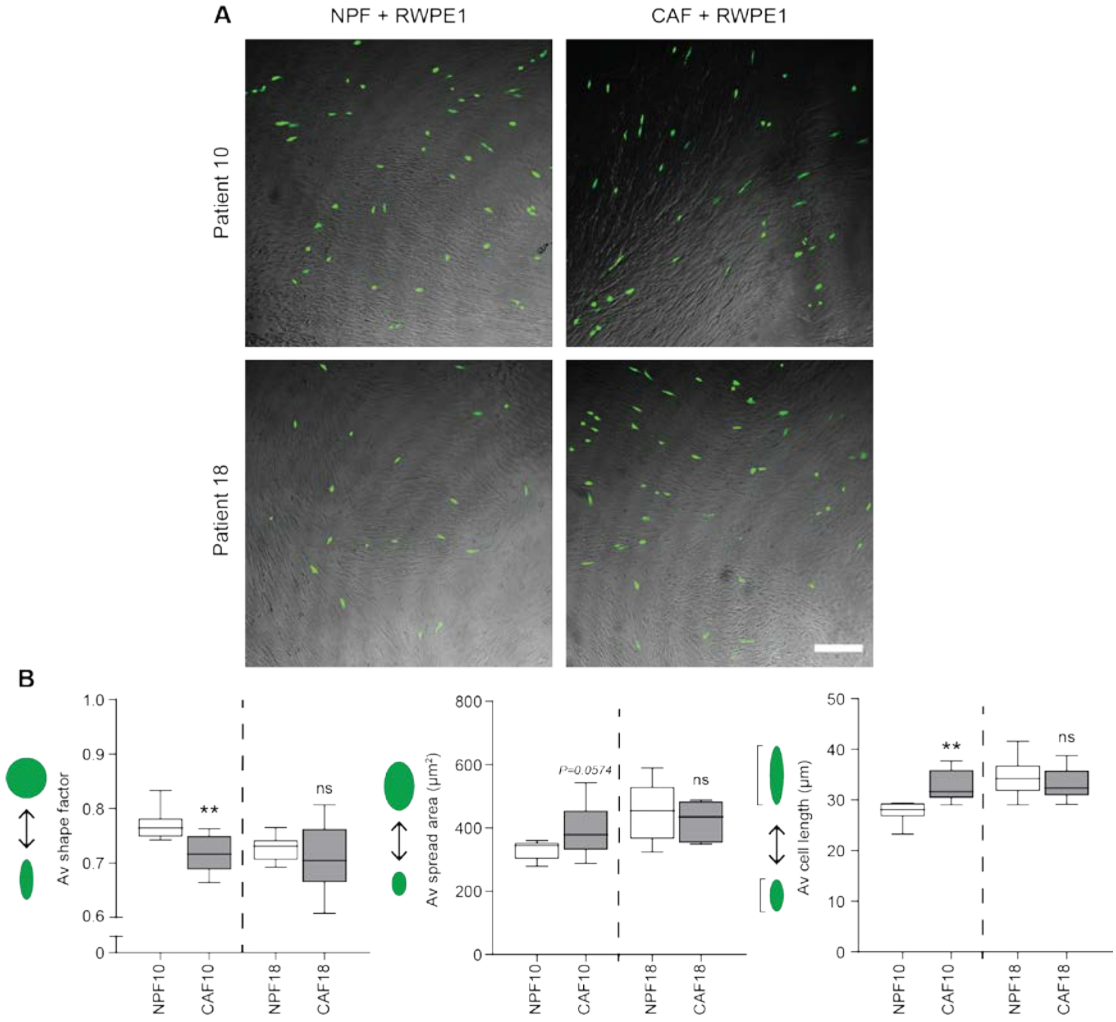

Figure S2
Concordance between *in vitro* functional assays and DNA methylation profiles. Patient 10 was used as a representative positive control, with co-cultures displaying the expected morphological changes, unlike Patient 18. **a** Representative images of co-cultures between matched CAFs and NPFs from Patients 10 and 18 and RWPE-1 prostate epithelial cells, which are labelled in green. RWPE-1 cells adopt more elongated, spindle-shaped morphology when co-cultured with CAF10 versus NPF10, but this trend is not apparent in CAF18 versus NPF18. Scale bar equals 200 µm. **b** Quantitative image analysis of RWPE-1 morphology in co-cultures with CAFs and NPFs. There is a significant decrease in the average shape factor and a significant increase in the average cell length of RWPE-1 cells in CAF10 versus NPF10 co-cultures, indicative of a more migratory phenotype (***P*<0.01, T test, n=8 fields of view per co-culture). There is no significant change in these parameters between co-cultures with CAF18 versus NPF18. Schematics in green next to each graph indicate how changes in cell morphology (shape, spread area and length) are measured.

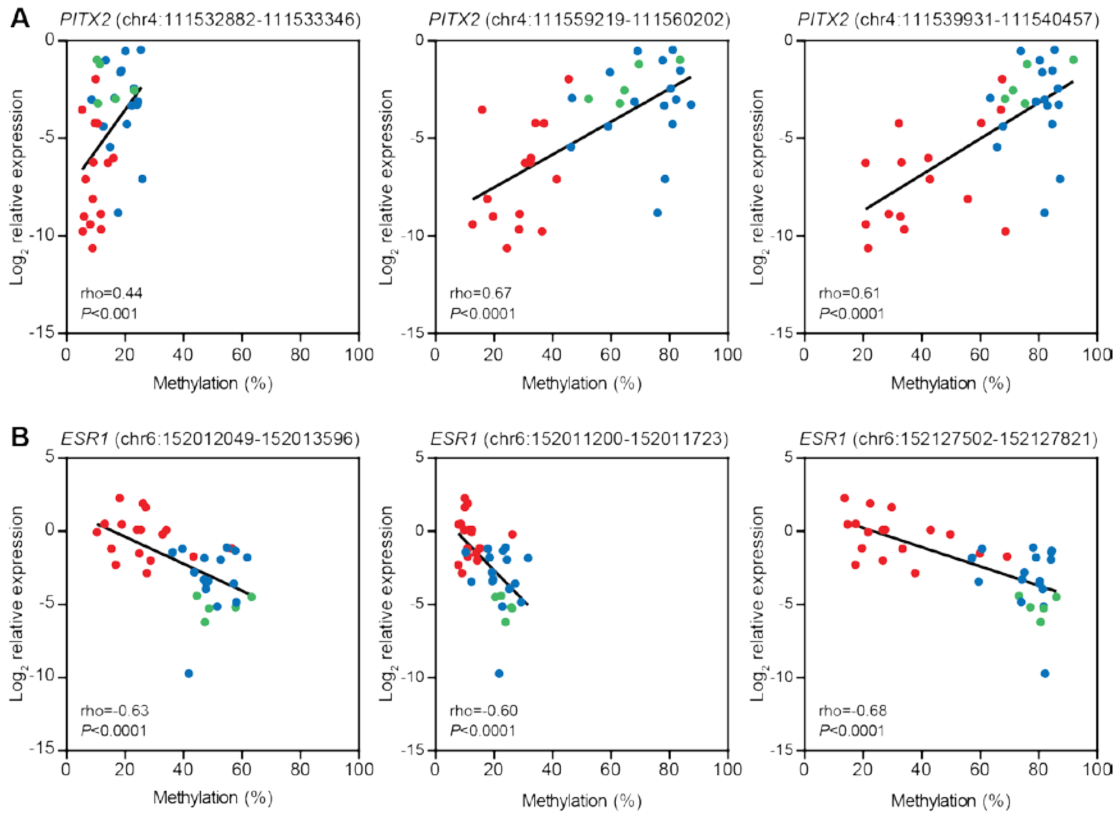

Figure S3

The correlation between methylation and mRNA abundance of CAF-DMRs. Plots show relative gene expression measured using qRT-PCR versus the percentage of DNA methylation measured using EPIC arrays for (**a**) *PITX2* and (**b**) *ESR1*. Both genes had three CAF-DMRs covered by probes on EPIC arrays. The co-ordinates of each CAF-DMR are shown and methylation values were averaged across EPIC probes within each CAF-DMR. Gene expression values below the detection limit are not shown. *P* and rho values are from Spearman correlations.

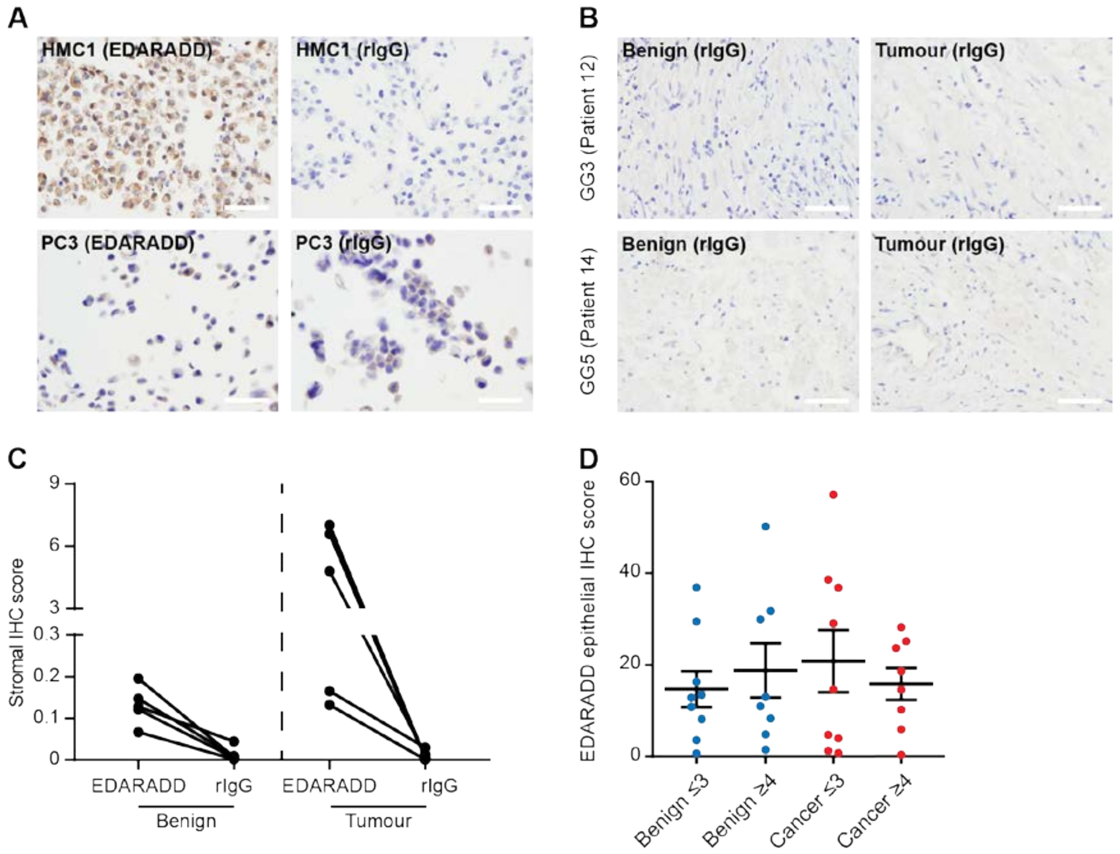

Figure S4

Controls and epithelial staining for EDARADD immunohistochemistry. **a** Images of EDARADD and rIgG immunoreactivity in cell pellets of HMC1 cells (EDARADD high) and PC3 cells (EDARADD low). **b** Representative images of immunoreactivity for rIgG in benign and tumour samples matching those stained for EDARADD in Figure 4c. **c** Plot of stromal IHC scores for benign and tumour samples stained with EDARADD and rabbit IgG control (rIgG). There was lower immunoreactivity with rIgG, including for the tumour samples with high levels of EDARADD. **d** Plot of average IHC scores (± SEM) for EDARADD staining in the epithelium of benign (blue) and tumour (red) tissue. There were no significant differences between any of the patient groups (One Way ANOVA with Tukey post-hoc analysis). All scale bars are 50 μm.

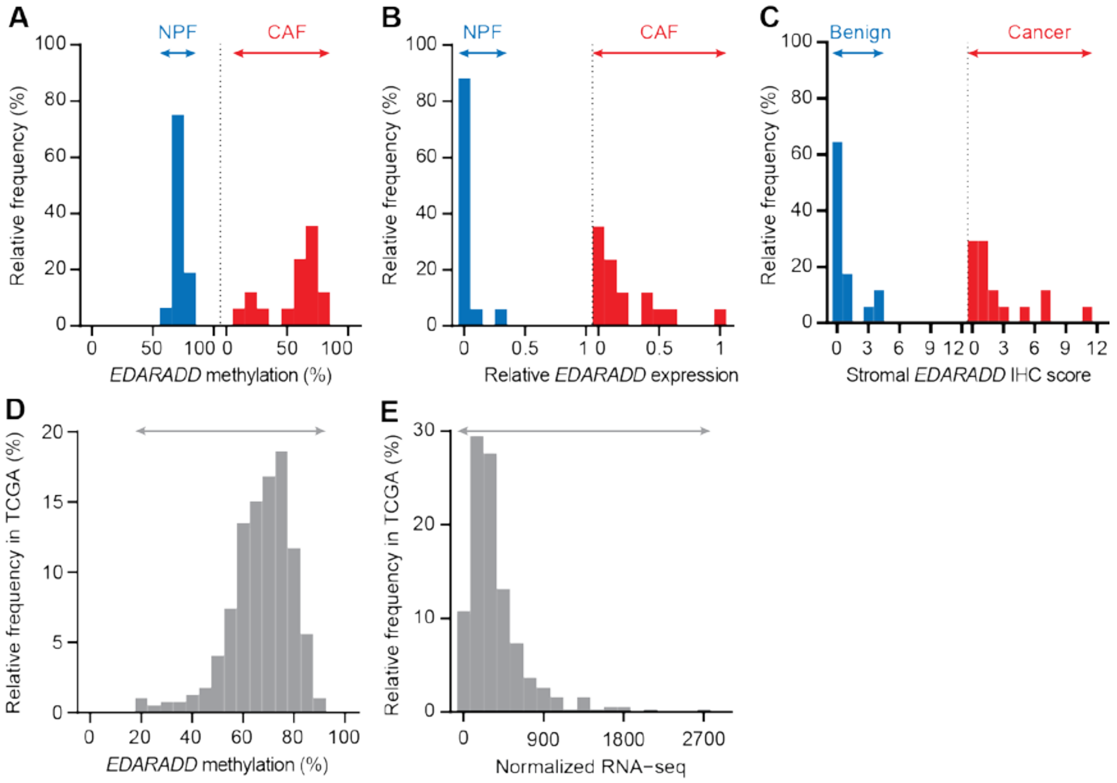

Figure S5

A subset of samples exhibit large differences in *EDARADD* methylation, expression and stromal staining. **a-b** Histograms showing the distribution of NPF and CAF samples according to the relative frequency of *EDARADD* (**a**) methylation and (**b**) relative expression levels. **c** Histogram showing the distribution of benign and tumour samples (matching NPFs and CAFs) based on the relative frequency of the stromal EDARADD IHC scores. **d-e** Histograms showing the distribution of TCGA tumour samples based on the relative frequency of *EDARADD* (**d**) methylation levels and (**e**) normalized RNA abundance from RNA-seq. Arrows denote the span of values in each panel.

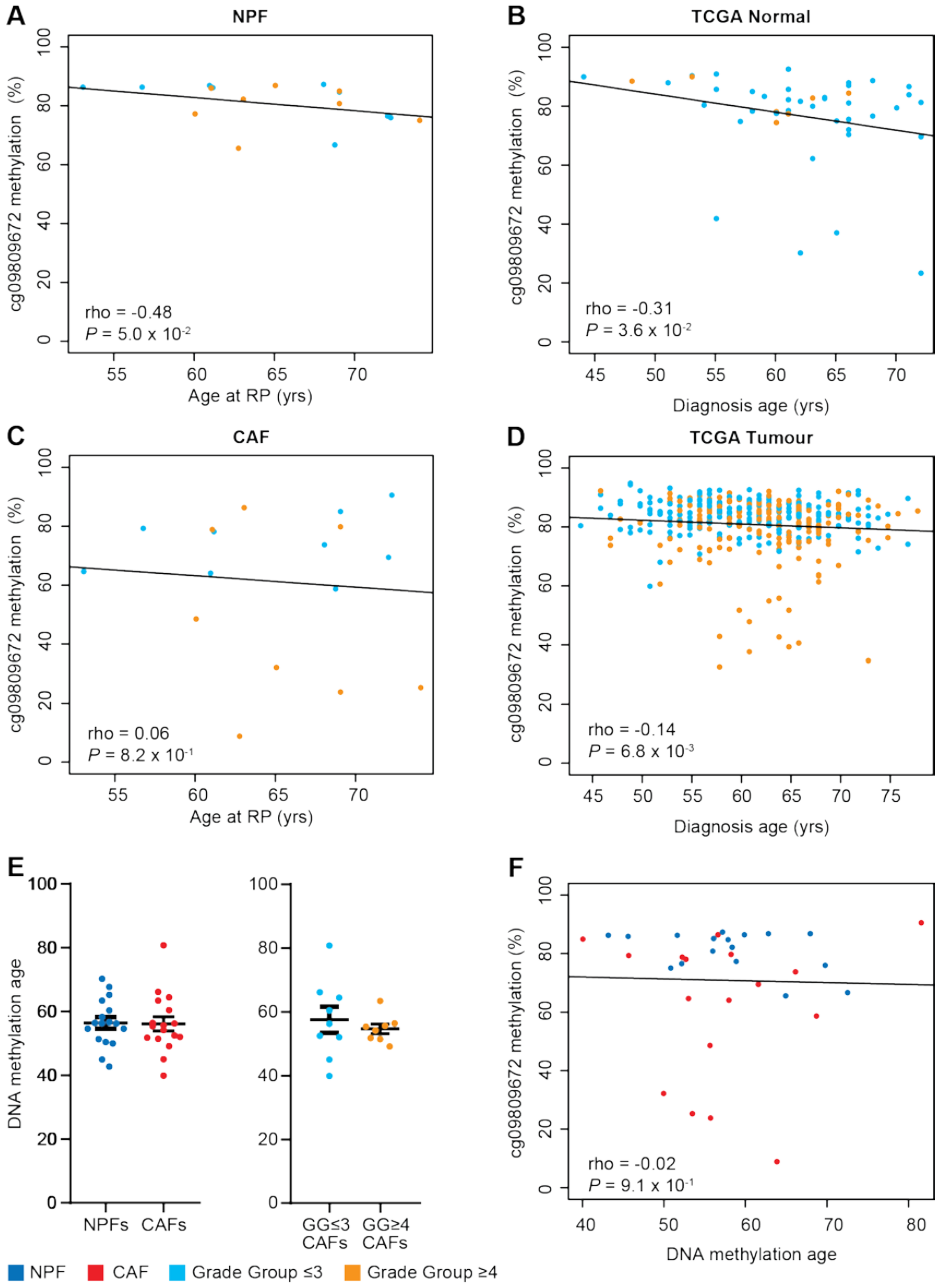

Figure S6

EDARADD hypomethylation is associated with age in non-malignant prostate tissue, but does not signify a more widespread DNA methylation aging phenotype in cancer. **a-d** Scatter plots comparing cg09809672 DNA methylation versus patient age for (**a**) NPFs, (**b**) non-malignant tissue from TCGA, (**c**) CAFs, and (**d**) tumour tissue from TCGA. Samples from patients with GG≤3 tumours are shown in light blue, while samples from patients with GG≥4 are in orange, RP = Radical Prostatectomy. Rho and *P* values are from Spearman's correlations. **e** Plots showing the average (± SEM) calculated DNA methylation age based on the Horvath signature for NPFs versus

CAFs and GG≤3 CAFs versus GG≥4 CAFs. There are no significant differences between groups (paired T test for NPF vs CAF, unpaired T test for GG≤3 CAFs versus GG≥4 CAFs). **f** Scatter plot of cg09809672 DNA methylation in CAFs and NPFs showing no significant correlation with the calculated DNA methylation age from the Horvath signature (Spearman’s correlation).

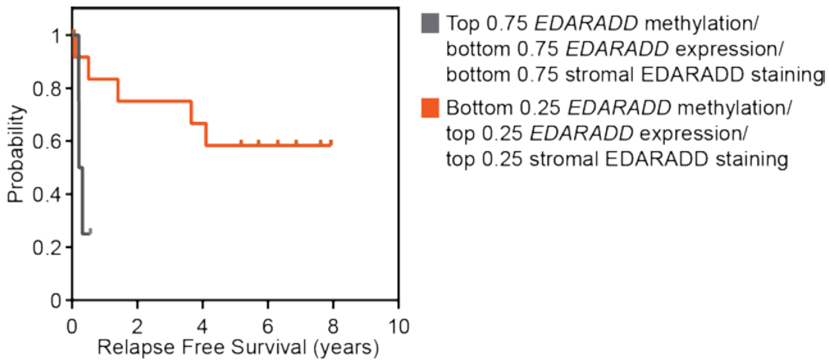

Figure S7
EDARADD methylation, expression and IHC staining is associated with poor relapse free survival. Kaplan Meier plot of relapse free survival for patients in the lowest quartile of *EDARADD* methylation and top quartile of expression (orange) versus the rest of the cohort (grey). The Kaplan Meier plots for methylation, expression and IHC data are the same, because the same patients were in the lowest quartile of *EDARADD* methylation and the highest quartile of *EDARADD* expression or stromal EDARADD staining. For methylation, the Cox HR = 0.96 (0.931-0.998), *P*=0.040. For expression, the Cox HR = 5.49 (0.29-47.93), *P*=0.123. For stromal EDARADD staining, the Cox HR = 1.26 (1.021-1.544), *P*=0.031.
